# Proteolytic cleavage of transmembrane cell adhesion molecule L1 by extracellular matrix molecule Reelin is important for mouse brain development

**DOI:** 10.1038/s41598-017-15311-x

**Published:** 2017-11-10

**Authors:** David Lutz, Ahmed Sharaf, Dagmar Drexler, Hardeep Kataria, Gerrit Wolters-Eisfeld, Bianka Brunne, Ralf Kleene, Gabriele Loers, Michael Frotscher, Melitta Schachner

**Affiliations:** 1 Institute for Structural Neurobiology, University Medical Center Hamburg-Eppendorf, Martinistr. 52, 20246 Hamburg, Germany; 2 Institute for Biosynthesis of Neural Structures, Center for Molecular Neurobiology, University Medical Center Hamburg-Eppendorf, Martinistr. 52, 20246 Hamburg, Germany; 3 Keck Center for Collaborative Neuroscience and Department of Cell Biology and Neuroscience, Rutgers University, 604 Allison Road, Piscataway, NJ 08854 USA; 4 Center for Neuroscience, Shantou University Medical College, 22 Xin Ling Road, Shantou, Guandong 515041 China

## Abstract

The cell adhesion molecule L1 and the extracellular matrix protein Reelin play crucial roles in the developing nervous system. Reelin is known to activate signalling cascades regulating neuronal migration by binding to lipoprotein receptors. However, the interaction of Reelin with adhesion molecules, such as L1, has remained poorly explored. Here, we report that full-length Reelin and its N-terminal fragments N-R2 and N-R6 bind to L1 and that full-length Reelin and its N-terminal fragment N-R6 proteolytically cleave L1 to generate an L1 fragment with a molecular mass of 80 kDa (L1-80). Expression of N-R6 and generation of L1-80 coincide in time at early developmental stages of the cerebral cortex. Reelin-mediated generation of L1-80 is involved in neurite outgrowth and in stimulation of migration of cultured cortical and cerebellar neurons. Morphological abnormalities in layer formation of the cerebral cortex of *L1*-deficient mice partially overlap with those of Reelin-deficient *reeler* mice. *In utero* electroporation of L1-80 into *reeler* embryos normalised the migration of cortical neurons in *reeler* embryos. The combined results indicate that the direct interaction between L1 and Reelin as well as the Reelin-mediated generation of L1-80 contribute to brain development at early developmental stages.

## Introduction

During nervous system development, the cell adhesion molecule L1^1,2^ plays crucial roles in proliferation, migration and survival of neural cells, and L1 participates in neuritogenesis and axonal outgrowth, guidance, pathfinding and fasciculation as well as in myelination and synaptogenesis^3–6^. L1-deficient mice show severe malformations and malfunctions of the nervous system^7,8^. In humans, mutations in L1 are associated with the L1 syndrome which comprises a spectrum of mild to severe congenital X-linked developmental disorders^6,9^. The L1 syndrome is characterised by developmental and mental retardation, intellectual deficits, hydrocephalus with stenosis of the aqueduct of Sylvius, corpus callosum agenesis, adducted thumbs, shuffling gait, aphasia and spastic paraplegia^6,9^.

The protein backbone of L1 consists of a C-terminal intracellular tail, a transmembrane domain and an extracellular part composed of 6 immunoglobulin-like (Ig) and 5 fibronectin type III (FN III) domains. Full-length L1 can undergo proteolytic ectodomain shedding to release soluble fragments and to generate transmembrane fragments^10–12^, which have been implicated in distinct L1 functions^10–20^. Cleavage of L1 within the third FN III domain by the serine proteases trypsin^13^, proprotein convertase PC5a^10^, and plasmin^14–16^ leads to promotion of neurite outgrowth and stimulates migration of neuronal cells.

The extracellular matrix protein Reelin controls the sequential lamination of the cerebral cortex, and the cortical layers are disorganised in the natural Reelin-deficient mutant *reeler* ^21–27^. Since Reelin was found to show serine protease activity cleaving extracellular matrix proteins^28^ and since Reelin-mediated cleavage of the cell surface glycoprotein Caspr had been reported to alter neurite outgrowth^29^, we investigated whether Reelin cleaves L1 and whether the serine protease activity of Reelin affects L1-dependent functions in the nervous system.

Here, we show that full-length Reelin and its fragments N-R2 and N-R6 bind to L1. We also show that full-length Reelin and its fragment N-R6 proteolytically cleave L1 within amino acids 840–845, and that serine_1283_ in Reelin is required for the cleavage of L1 by Reelin. Furthermore, we show that the protease activity of Reelin is required for L1-mediated neurite outgrowth and neuronal migration, and we provide evidence that cleavage of L1 by Reelin affects early stages of brain development.

## Results

### Reelin proteolytically cleaves cell adhesion molecule L1

**Figure 1:**
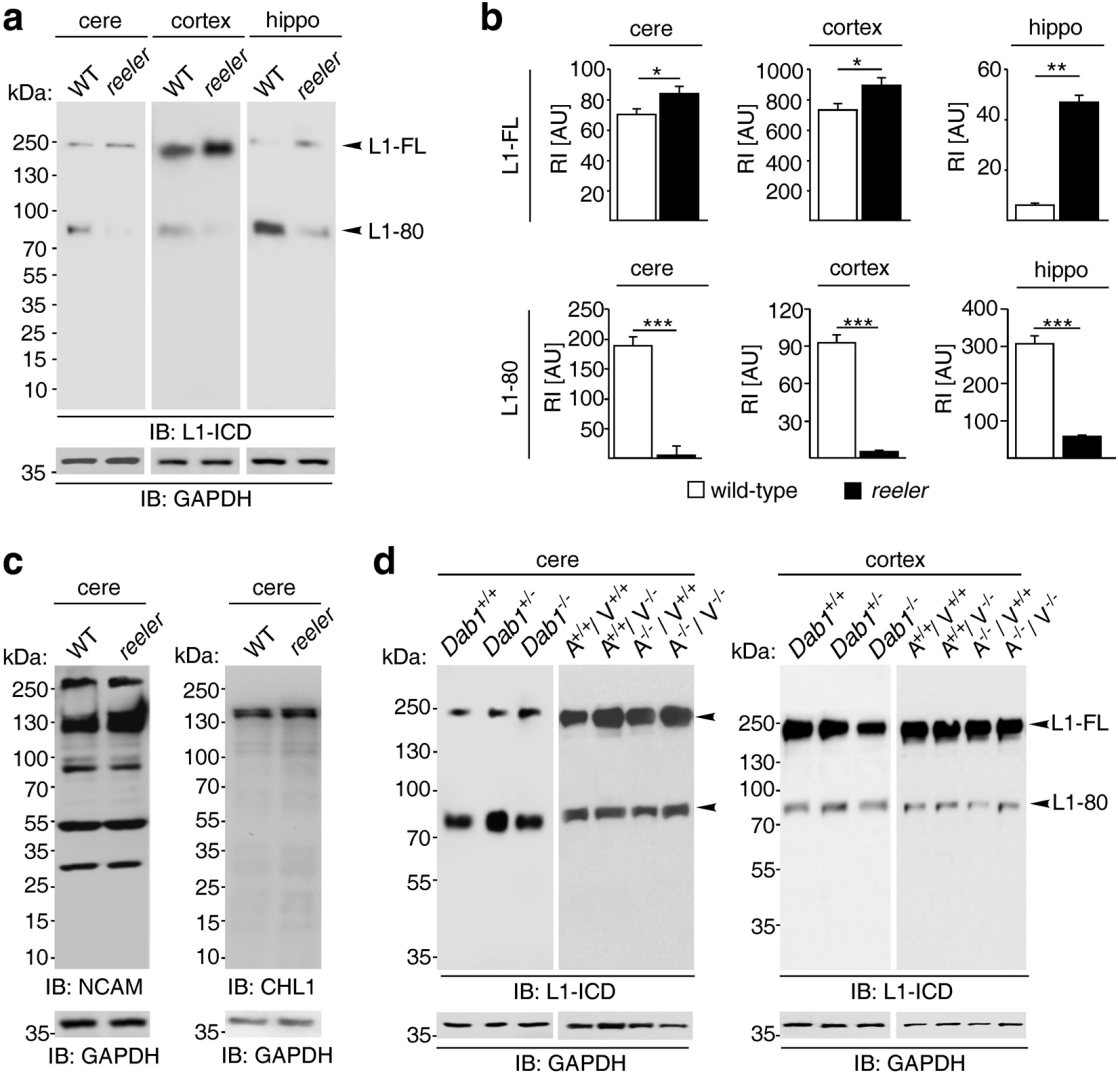
L1-80 levels are decreased in *reeler* mice. (**a**) Immunoblot analysis of homogenates from cerebellum (cere), cerebral cortex (cortex) and hippocampus (hippo) of 6-day-old wild-type (WT) and *reeler* mice with an antibody against the intracellular L1domain (L1-ICD). L1-FL: full-length L1. (**b**) Quantification of L1-FL and L1-80 levels in homogenates from cerebellum, cerebral cortex and hippocampus of wild-type and *reeler* mice. Mean values + SEM from 6 independent experiments and differences between groups are shown (*p < 0.05, **p < 0.01, ***p < 0.005; two-tailed t-test). RI: relative intensity in arbitrary units (AU). (**c**) Unaltered NCAM and CHL1 expression levels in cerebellar homogenates from wild-type (WT) and *reeler* mice. (**d**) Unaltered L1-80 levels in cerebellar and cerebral cortex homogenates from wild-type (*Dab1* ^+/+^), heterozygous (*Dab1* ^+/−^), and *Dab1* knock-out (*Dab1* ^−/−^) mice, and in homogenates from *ApoER2-VLDLR* double knock-out (A^−/−^V^−/−^), heterozygous (A^+/+^V^−/−^, A^−/−^V^+/+^) and wild-type (A^+/+^V^+/+^) littermates. (**a,c,d**) Representative immunoblots out of 6 independent experiments are shown and display all L1, CHL1 and NCAM forms. GAPDH antibody was used to control loading and only the regions of the blots with GAPDH bands are shown.

To determine if Reelin cleaves L1 we probed tissue from detergent-solubilised brain regions of postnatal *reeler* mice and wild-type littermates for L1-fragments using immunoblot analysis with antibody 172, which recognises the intracellular L1 domain. In the cerebellum, cerebral cortex and hippocampus of *reeler* mice, the expression of a proteolytic 80 kDa L1 fragment (L1–80) was decreased when compared to its wild-type levels (Fig. 1a,b). The protein expression of the close homolog of L1 (CHL1) and the neural cell adhesion molecule NCAM were similar in the cerebellum, cerebral cortex and hippocampus of wild-type and *reeler* mice (Fig. 1c and Supplementary Fig. S1a,b). Since double knock-out of the Reelin receptors *ApoER2* and *VLDLR* or deficiency of *Dab1*, which is the downstream signal transducer of ApoER2 and VLDLR, lead to a *reeler*-like phenotype^30–34^, we next analysed the expression of L1 proteolytic fragments in brain tissue homogenates from *Dab1*-deficient and *ApoER2* and *VLDLR* single and double knock-out mice. We found that the L1 expression in the cerebral cortex and cerebellum of these mice were similar to those in wild-type mice and heterozygous littermates (Fig. 1d and Supplementary Fig. S2), indicating that Reelin is involved in the proteolytic processing of L1 to generate L1-80 independently of the signalling cascade via ApoER2, VLDLR and Dab1.

**Figure 2:**
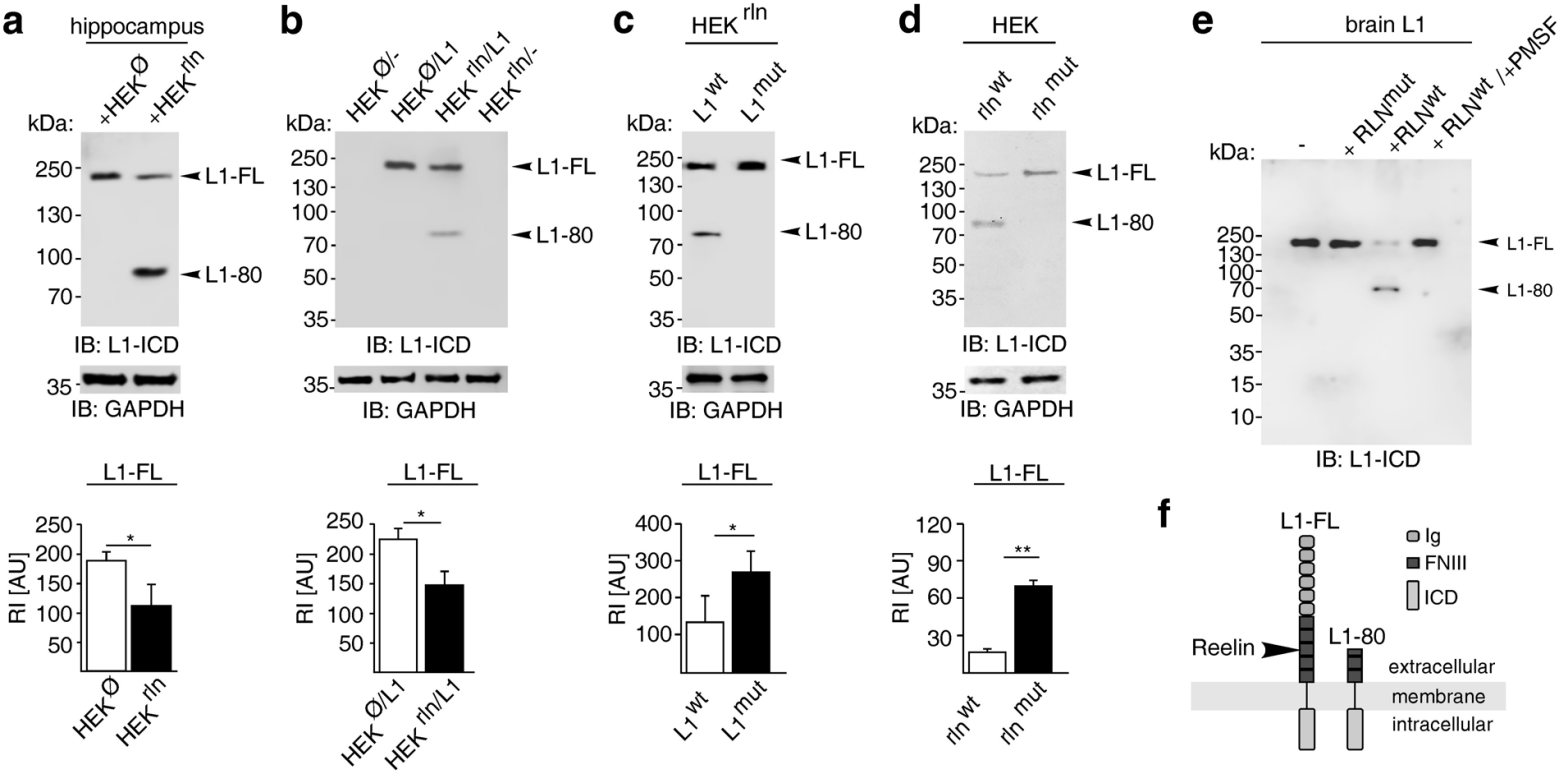
Reelin cleaves L1. (**a**) Freshly homogenized *reeler* hippocampus was treated with supernatants from HEK cells expressing (HEK^rln^) or lacking (HEK^Ø^) Reelin. (**b**) HEK^rln^ and HEK^Ø^ cells were transfected with wild-type L1 (L1) or mock DNA (−). (**c**) HEK^rln^ cells were transfected with wild-type (L1^wt^) or mutated (L1^mut^) L1. (**d**) HEK cells were co-transfected with wild-type L1 and wild-type Reelin (rln^wt^) or mutated Reelin (rln^mut^). (**a–d**) Representative immunoblots of cell lysates out of six independent experiments using an antibody against the intracellular L1 domain (L1-ICD) and the GAPDH antibody are shown. The cropped blots display all L1 forms or the GAPDH band. Mean values  + SEM from 6 independent experiments are shown for the levels of full-length L1 (L1-FL) and differences between groups are shown (*p < 0.05, **p < 0.0001; One-way ANOVA with Tukey’s Multiple Comparison Test). RI: relative intensity in arbitrary units (AU). (**e**) Purified brain L1 was incubated with heparin-purified wild-type (RLN^wt^) or mutated (RLN^mut^) Reelin protein in the absence or presence of the serine protease inhibitor PMSF (+PMSF) and then subjected to immunoblot analysis with L1 antibody recognising the intracellular domain (L1-ICD). (**f)** Schematic representation of L1 structure and cleavage by Reelin. Shown are the extracellular Ig and FN III domains, the transmembrane and intracellular domain (ICD).

Since Reelin has been described to cleave fibronectin^28^ and since L1 contains five FN III domains, we tested whether Reelin can generate L1-80 and analysed which structural motifs in L1 are recognised by Reelin to generate L1-80. Freshly homogenized hippocampus from newborn *reeler* mice was incubated either with supernatants from HEK cells which were transfected to express and secrete Reelin or from mock-transfected HEK cells. L1-80 was detected in the homogenates treated with Reelin-containing supernatant, but not in the homogenates treated with mock-supernatant devoid of Reelin (Fig. 2a). L1-80 was also detected in embryonic *reeler* cerebral cortex homogenates after treatment with Reelin-containing supernatant, but not after treatment with Reelin-lacking mock-supernatant (Supplementary Fig. S3). In additional experiments, Reelin and L1 were expressed in L1-lacking HEK cells by co-transfection, and cell lysates were subjected to immunoblotting using L1 antibody 172. HEK cells transfected with L1 alone and non-transfected cells were used for comparison. L1-80 was detectable only in lysates from L1- and Reelin-expressing cells (Fig. 2b). These findings suggest that Reelin cleaves full-length L1 to generate L1-80.

Since the third FN III domain of L1 is a substrate for several proteases generating L1-80^10,15^, we tested whether Reelin also cleaves L1 at the previously identified motif _840_RKHSKR_845_^10^. Therefore, Reelin-expressing HEK cells were transfected with wild-type *L1* or *L1* harbouring mutations in this motif by changing it to _840_SKHSSS_845_^10^. L1-80 was detected in lysates of HEK cells co-expressing Reelin and wild-type L1, but not in cells co-expressing Reelin and mutated *L1* (Fig. 2c). These results indicate that _840_RKHSKR_845_ represents the cleavage site for Reelin in L1. Given that murine Reelin contains a serine in its enzymatically active centre at position 1283 (GKS_1283_D)^28^, we examined whether exchanging serine against alanine at this position would affect proteolysis of L1 by Reelin. HEK cells expressing L1 were transfected either with wild-type Reelin or Reelin with the S/A_1283_ mutation and cell lysates were probed by immunoblot analysis with L1 antibody 172. L1-80 was found in lysates of HEK cells expressing wild-type, but not mutated Reelin (Fig. 2d). In further experiments, we incubated heparin-purified wild-type Reelin^35^ or heparin-purified Reelin harbouring the S/A_1283_ mutation (Supplementary Fig. S4) with L1 immunoaffinity-purified from mouse brain^2,11^. L1-80 was only generated in the presence of wild-type Reelin, but not in the presence of mutated Reelin or in the concomitant presence of wild-type Reelin and the serine protease inhibitor phenylmethylsulfonyl fluoride (Fig. 2e). The combined findings show that S_1283_ in Reelin is essential for Reelin’s serine protease activity and for the generation of L1-80 by Reelin (Fig. 2f).

### Protease activity of Reelin is required for L1-mediated neurite outgrowth and neuronal migration *in vitro*

**Figure 3:**
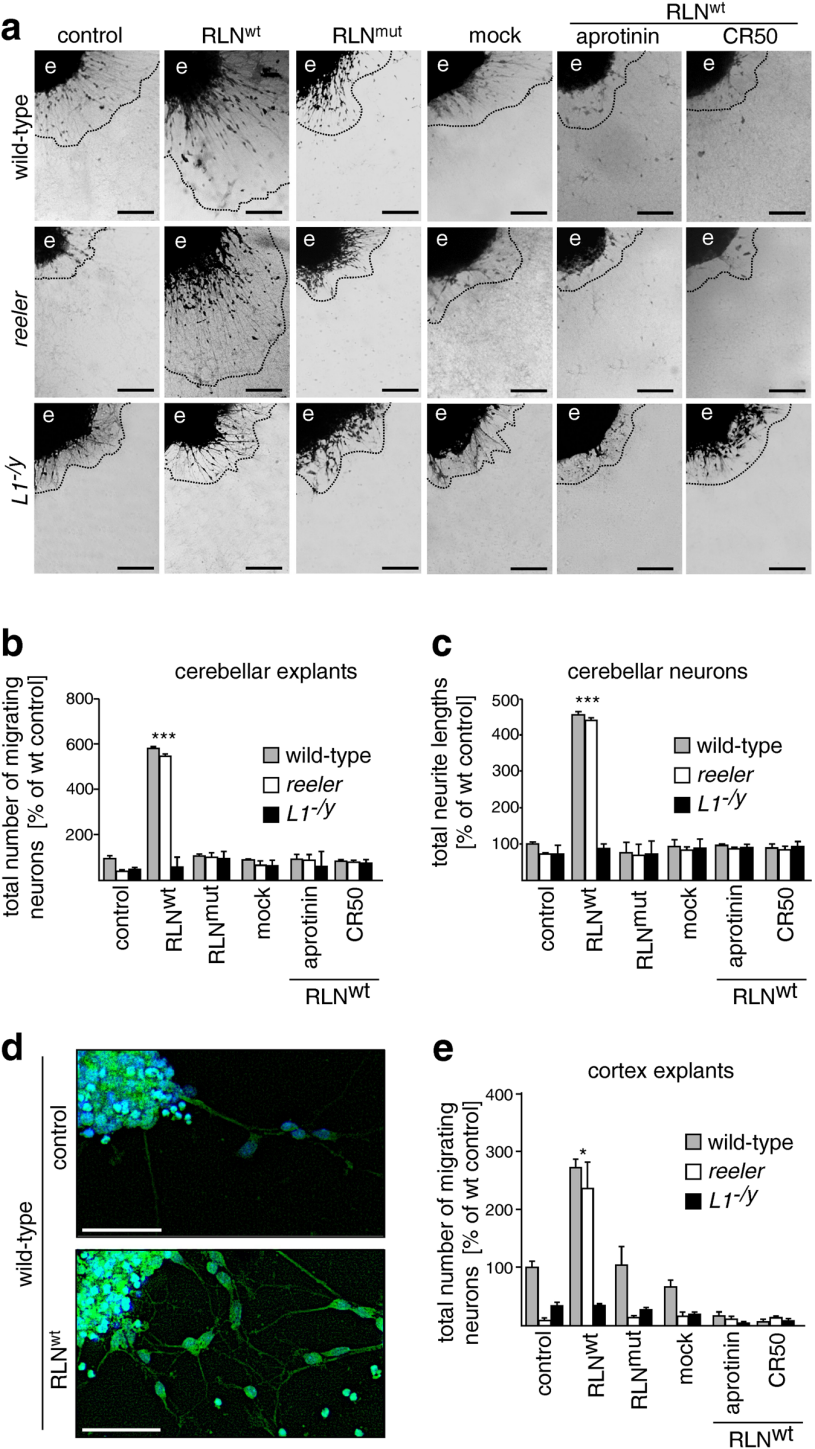
Reelin promotes L1-dependent neurite outgrowth and neuronal migration *in vitro* via its serine protease activity. Assessment of neuronal migration out of cerebellar explants from wild-type, *reeler* and *L1*-deficient (L1^−/y^) mice (**a**,**b**) and assessment of neurite lengths in dissociated wild-type, *reeler* and *L1*-deficient (L1^−/y^) cerebellar neurons (**c**) after treatment with purified wild-type Reelin (RLN^wt^) or with purified mutated Reelin (RLN^mut^) in the absence or presence of the serine protease inhibitor aprotinin or the Reelin-inhibiting antibody CR50. Untreated (control) and mock-treated neurons and explants were used as controls. (**a**) Representative images of explants after treatment are shown; scale bars: 100 µm. The dashed lines represent the outward borders of migration; e: explant. (**b**,**c**) Numbers of migrating cells and total neurite lengths were determined evaluating 30 equal-sized explants (**a**) or 100 neurons (**b**) per group from 3 independent experiments. Mean values + SEM are shown for numbers of migrating cells or total neurite lengths relative to values observed for untreated explants and cells from wild-type mice (set to 100%). Differences between groups are indicated (***p < 0.0001; One-way ANOVA with Tukey’s Multiple Comparison Test). (**d**,**e**) Cerebral cortex explants from wild-type, *reeler* and *L1*-deficient mice were treated with mutated Reelin (RLN^mut^) or wild-type Reelin (RLN^wt^) in the absence or presence of aprotinin or Reelin antibody CR50. Untreated (control) and mock-treated explants were used as controls. (**d**) Representative images of untreated and Reelin-treated wild-type explants stained for βIII-tubulin (green) are shown; scale bars: 50 µm. Nuclei are stained with DAPI (blue). (**e**) Numbers of migrating cells were determined evaluating 10 equal-sized explants per group from 3 independent experiments. Mean values + SEM are shown for numbers of migrating cells relative to values observed for untreated explants from wild-type mice (set to 100%). Differences between groups are indicated (*p < 0.05; One-way ANOVA with Holm-Sidak Multiple Comparison Test).

To investigate whether L1-mediated neuronal migration or neurite outgrowth depend on Reelin’s serine protease activity, wild-type, *reeler*, and *L1*-deficient dissociated cerebellar neurons or cerebellar explants were treated with purified wild-type Reelin alone or in the presence of the serine protease inhibitor aprotinin or the Reelin function-inhibiting antibody CR50^28,36,37^. Neurons and explants treated with Reelin carrying the S/A_1283_ mutation as well as mock-treated and untreated neurons and explants were investigated for comparison. Explants from *reeler* and *L1*-deficient mice showed reduced neuronal migration when compared to wild-type explants (Fig. 3a,b). Wild-type Reelin promoted neuronal migration from explants of *reeler* and wild-type mice, but not from explants of *L1*-deficient mice, when compared to explants treated with mutated, proteolytically inactive Reelin or to mock-treated or non-treated explants (Fig. 3a,b). In the presence of the function-inhibiting Reelin antibody CR50 or the serine protease inhibitor aprotinin, neuronal migration from explants of *reeler* and wild-type mice was not enhanced by wild-type Reelin. Application of aprotinin or of Reelin antibody CR50 had no effect on migration from explants of *L1*-deficient mice (Fig. 3a,b). Since it has been shown by βIII-tubulin immunostaining that the thin processes around the explant core are of neuronal origin^12^, we conclude that in this assay the neuronal migration takes place along neurites and not along radial glial fibers. Application of wild-type Reelin to *reeler* and wild-type neurons led to enhanced total neurite lengths, whereas application of wild-type Reelin to *L1*-deficient neurons did not stimulate the extension of neurites (Fig. 3c). In the presence of Reelin antibody CR50 or aprotinin, wild-type Reelin did not enhance of neurite outgrowth from *reeler* and wild-type neurons (Fig. 3c). Application of aprotinin and CR50 together with Reelin did not affect neurite outgrowth in *L1*-deficient neurons (Fig. 3c). These results indicate that L1-mediated neurite outgrowth and neuronal migration in the cerebellum depend on the serine protease activity of Reelin.

To examine whether neuronal migration along neurites in the cerebral cortex also depends on the serine protease activity of Reelin, cortical explants from wild-type, *reeler* and *L1*-deficient mice were treated as described above for treatment of cerebellum explants and were then immunostained for βIII-tubulin. In wild-type explants, Reelin promoted neuronal migration along βIII-tubulin-positive neurites (Fig. 3d). Similarly, Reelin promoted neuronal migration along neurites on *reeler* explants, but not on explants from *L1*-deficient mice (Fig. 3e). In comparison to untreated wild-type explants, untreated *reeler* and *L1*-deficient explants showed reduced neuronal migration (Fig. 3e). Mock treatment and application of mutated Reelin did not result in promotion of neuronal migration (Fig. 3e). Reelin antibody CR50 and aprotinin inhibited the Reelin-promoted neuronal migration from *reeler* and wild-type explants (Fig. 3e). Aprotinin and CR50 did not affect the migration from explants of *L1*-deficient mice (Fig. 3e). These results indicate that the serine protease activity of Reelin is required for L1-mediated neuronal migration in the cerebral cortex.

### Reelin interacts with L1

**Figure 4:**
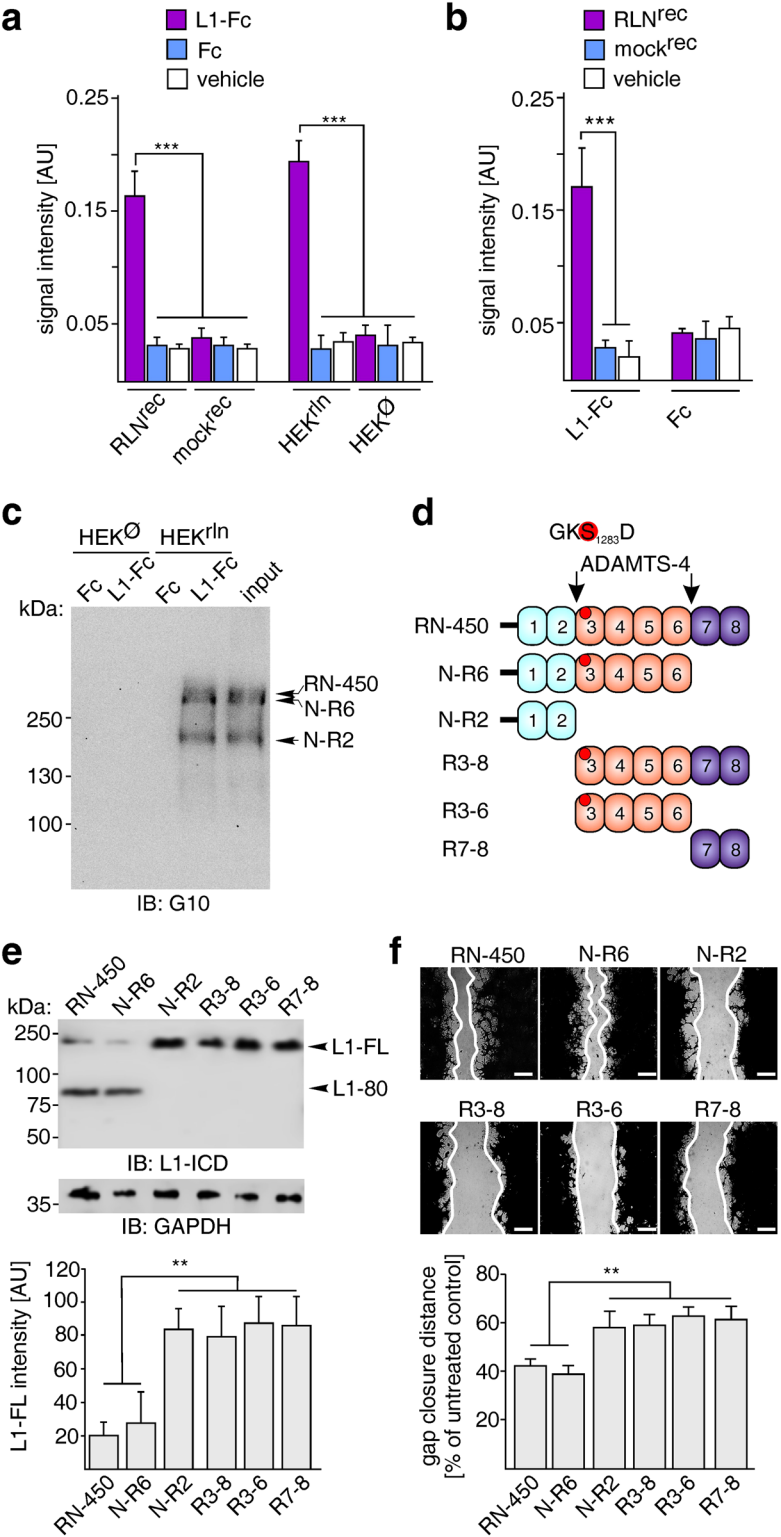
Full-length Reelin and Reelin fragment N-R6 interact with L1 and cleave L1 to accelerate migration *in vitro*. (**a**) ELISA using substrate-coated L1-Fc, Fc or vehicle solution (phosphate buffer saline, pH 7.3) and purified Reelin protein (RLN^rec^), mock-purified solution (mock^rec^) or cell culture supernatants from Reelin-expressing (HEK^rln^) or Reelin-lacking (HEK^Ø^) HEK cells. (**b**) ELISA using substrate-coated purified Reelin protein (RLN^rec^), mock-purified solution (mock^rec^) or vehicle solution and L1-Fc or Fc. (**a**,**b**) Mean values + SEM for binding are shown (***p < 0.0001; One-way ANOVA with Tukey’s Multiple Comparison Test from 3 independent experiments with triplicates). (**c**) Pull-down with L1-Fc and Fc using supernatants from Reelin-expressing (HEK^rln^) and Reelin-lacking (HEK^Ø^) HEK cells. Supernatant from Reelin-expressing HEK cells was used as input control and Reelin antibody G10 was used for detection. A representative immunoblot out of 3 independent experiments with Reelin-positive bands is shown and full-length Reelin (RN-450) and its fragments N-R6 and N-R2 are indicated. (**d**) Structure of Reelin and its proteolytic fragments after N- and C-terminal processing by ADAMTS-4. The enzymatically decisive serine residue in the GKS_1283_D sequence in murine Reelin is indicated by a red spot. (**e**) Lysates from co-transfected HEK cells expressing full-length Reelin (RLN-450) or Reelin fragments N-R6, N-R2, R3-6, R3-8 or R7-8 were subjected to immunoblot analysis with L1 antibody against the intracellular domain (L1-ICD) and GAPDH antibody to control loading. Representative immunoblots out of 6 independently experiment are shown and display all L1 forms and the GAPDH bands. Quantification of full-length L1 (L1-FL) levels (AU: arbitrary units) is shown in the lower panel. Mean values + SEM from 6 independent experiments are shown and differences between groups are indicated (**p < 0.001; One-way ANOVA with Tukey’s Multiple Comparison Test). (**f**) Images of scratches in monolayers of co-transfected HEK cells expressing L1 and full-length Reelin or one of the indicated Reelin fragments. Gap borders are highlighted with continuous white lines; scale bars: 50 µm. Mean values + SEM from 3 independent experiments with triplicates per group are shown for gap closure after scratching. Differences are indicated (**p < 0.005; One-way ANOVA with Tukey’s Multiple Comparison Test).

Since the N-terminus of Reelin binds to RGD motifs^38^, which are present in L1^15^, we investigated whether L1 interacts with Reelin or Reelin fragments^39–41^. ELISA was performed with purified recombinant Reelin or Reelin-containing HEK cell supernatants as coated substrates and with addition of L1-Fc or Fc. Coatings with supernatants lacking Reelin, mock supernatants or vehicle solution were used as negative controls. In turn, ELISA was also performed using substrate-coated L1-Fc or Fc and applying purified recombinant Reelin, Reelin-containing HEK cell supernatant, mock-purified cell culture supernatant or vehicle solution. L1-Fc showed pronounced binding to substrate-coated recombinant Reelin and to Reelin-containing cell supernatant, whereas Fc, mock-treated supernatant or vehicle solution did not show significant binding (Fig. 4a). Similarly, recombinant Reelin and Reelin-containing HEK cell supernatants bound to substrate-coated L1-Fc, but not to Fc, whereas the mock-purified cell culture supernatant or vehicle solution did not bind to L1-Fc or Fc (Fig. 4b). L1-Fc and Fc were also used for pull-down assays with supernatants of Reelin-transfected and non-transfected HEK cells. Immunoblot analysis with Reelin antibody G10 revealed that L1-Fc, but not Fc, precipitated full-length Reelin and the Reelin fragments N-R6 and N-R2 from Reelin-containing HEK cell supernatants, but not from Reelin-lacking HEK cell supernatants (Fig. 4c). The combined results indicate that L1 associates with Reelin and its fragments N-R6 and N-R2.

### Reelin and its N-terminal fragment N-R6 are proteases in the embryonic, early developing cerebral cortex and generate L1-80

N- and/or C-terminal cleavage of Reelin by ADAMTS-4 (for sequences of Reelin fragments, see ref.^40^) yields two N-terminal fragments (N-R2 and N-R6), two fragments containing the central part of Reelin (R3-6 and R3-8), and one fragment containing the C-terminus (R7-8) (Fig. 4d). Thus, we analysed whether only full-length Reelin cleaves L1 or whether one of the Reelin fragments could also cleave L1 to generate L1-80. Full-length L1-expressing HEK cells were transfected with full-length Reelin or Reelin fragments N-R6, R3-8 or R3-6 which carry the GKS_1283_D motif. Cells expressing the Reelin fragments N-R2 or R7-8 which lack the GKS_1283_D motif were used as controls. L1-80 was detectable in lysates of HEK cells co-expressing full-length L1, full-length Reelin or the N-R6 Reelin fragment, but not in lysates of HEK cells co-expressing L1 and the Reelin fragments N-R2, R3-8, R3-6 or R7-8 (Fig. 4e), suggesting that only full-length Reelin and N-R6 generate L1-80. These results indicate that the N-terminal proteolytic processing of Reelin^41^ generates the fragments N-R2, which does not contain GKS_1283_D, and the proteolytically inactive R3-8 and R3-6.

We next studied which Reelin fragment affects L1-dependent cell migration in an *in vitro* scratch injury assay using transfected HEK cells. It was not possible to perform this assay with primary cultures of neurons, since these neurons do not form uniform monolayers and do not survive upon scratching. Moreover, the low transfection efficiency of neurons precludes their usage in this experimental setup. It was plausible to use HEK cells, because they use similar signalling pathways for migration as neurons do during development, i.e. they respond to Reelin with enhanced migration when expressing L1. Widths of gaps were measured 16 h after scratching of monolayers of HEK cells which were transfected to co-express L1 and full-length Reelin or Reelin fragments N-R6, N-R3, R3-8, R3-6 or R7-8. Only the N-R6 fragment and full-length Reelin, but not the N-R2, R3-8, R3-6 and R7-8 fragments, enhanced the gap closure (Fig. 4f), suggesting that the protease activities of N-R6 and full-length Reelin are required for L1-mediated cell migration.

**Figure 5:**
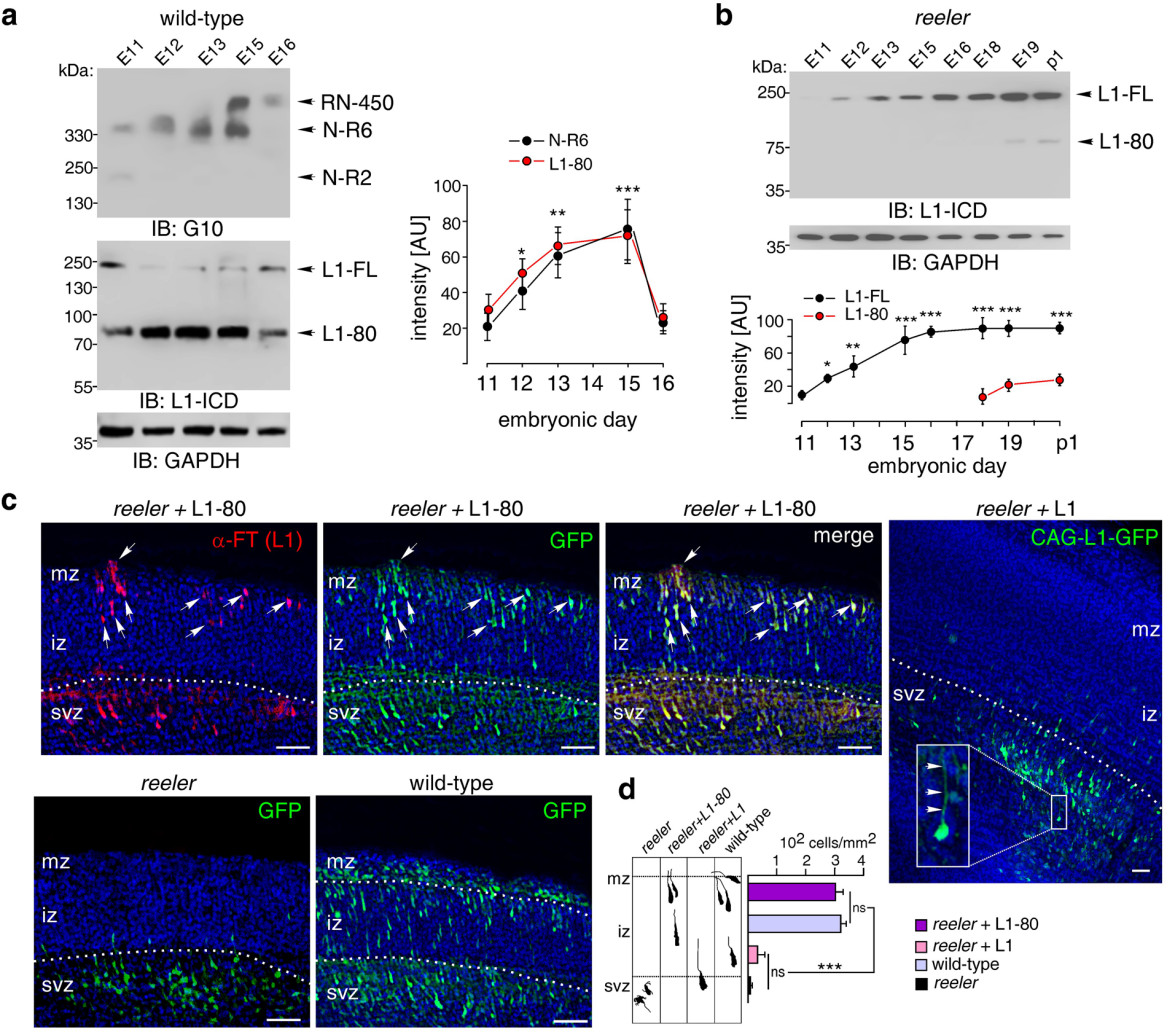
Interaction between Reelin and L1 and functional roles of their fragments in the developing brain. (**a,b**) Immunoblot analysis of Reelin and L1 expression in wild-type and *reeler* cerebral cortex homogenates at different embryonic stages using the Reelin antibody (G10) or an antibody against the intracellular L1 domain (L1-ICD). GAPDH antibody was used to control loading. Representative immunoblots out of 6 independent experiments are shown and display all L1 and Reelin forms and the GAPDH bands. Quantifications of N-R6, full-length L1 (L1-FL) and L1-80 levels are given in arbitrary units (AU). Mean values ± SEM from 6 independent experiments and differences relative to the values at embryonic day 11 are shown (*p < 0.05; **p < 0.001; ***p < 0.0001; One-way ANOVA with Tukey’s Multiple Comparison Test). (**c**) L1-80 carrying a flag-tag was electroporated together with CAG-GFP into *reeler* embryos at embryonic day 13.5. Sixty hours after *in utero* electroporation, cerebral cortex was analysed for L1-80-expressing neurons using an antibody against the flag-tag (α-FT) and a fluorescently labelled secondary antibody (red). High numbers of GFP-positive (green) and L1-80-positive (red) neurons were found in the upper layers of the cerebral cortex from *reeler* mice after electroporation with L1-80 (white arrows) and in the cortex from wild-type mice. After electroporation with CAG-GFP coding for full-length L1 or with CAG-GFP alone, GFP-positive neurons were retained in the subventricular zone (svz). Inset: L1 promotes elongation of the leading processes of some electroporated neurons. The marginal zone (mz) in cortices of *reeler* mice contains more cells than observed in wild-type mice. White dashed lines indicate the border between the svz and intermediate zone (iz) and between iz and mz. Nuclear staining with DAPI (blue) is shown. Scale bars: 50 µm. (**d**) Schematic representation of migration behaviour of *reeler* neurons electroporated with L1-80 or full-length L1 in comparison to *reeler* and wild-type neurons. Assessment of numbers of electroporated neurons within the iz and mz area was performed. Mean values + SEM from 6 independent experiments are shown for the cell density in the iz-mz-area and differences between groups are indicated (ns: not significant; ***p < 0.0001; One-way ANOVA with Tukey’s Multiple Comparison Test).

Since full-length Reelin and the N-R6 fragment cleave L1, we investigated by immunoblot analysis, whether expression of full-length Reelin and/or its N-R6 fragment coincides with the generation of L1-80 at different developmental stages in wild-type cerebral cortex homogenates. Levels of N-R6 and L1-80 were increased between embryonic days 12 and 15. N-R6 levels were already detected at embryonic day 11, but levels of full-length Reelin were only detected at embryonic days 15 and 16 (Fig. 5a). These results suggest that during embryonic development of the cerebral cortex, Reelin is rapidly processed to N-R6 which generates L1-80 at early developmental stages. Moreover, the findings suggest that the N-terminal processing of Reelin abolishes its cleavage activity and limits the generation of L1-80 during embryonic development.

It should be noted that L1 is cleaved by other serine proteases^10–12^, which, similarly to Reelin, cleave L1 at amino acids 840-845. To examine whether Reelin and/or its N-R6 fragment are responsible for the generation of L1-80 in the early cortical development, we measured L1-80 expression levels in *reeler* cerebral cortex at different embryonic stages. L1-80 was not detectable during embryonic corticogenesis in *reeler* mice and became detectable at low levels only at later stages of corticogenesis (Fig. 5b). These results implicate Reelin as serine protease in the embryonic cerebral cortex acting on L1 to produce L1-80.

To investigate whether L1-80 would normalise migration of *reeler* neurons during embryonic development of the *reeler* cerebrum, we electroporated L1-80 into the cerebral cortex of *reeler* embryos *in utero* at embryonic day 13.5 using a flag-tagged L1-80 plasmid in combination with a CAG-GFP plasmid for visualisation of the electroporated neurons. *Reeler* and wild-type embryos electroporated with CAG-GFP alone were used for comparison. Sixty hours after electroporation, L1-80-expressing neurons were identified as GFP-positive cells which were stained with the antibody recognising the flag-tag of L1-80 (Fig. 5c). Many double-labelled neurons had reached the *reeler* marginal zone as similarly observed for GFP-electroporated wild-type neurons (Fig. 5c,d). GFP-electroporated *reeler* neurons were misoriented and retained in the subventricular zone (Fig. 5c,d). In contrast to L1-80, full-length L1 did not promote migration of *reeler* neurons (Fig. 5c,d). Of note, some L1-electroporated neurons had elongated leading processes (Fig. 5c,d). These results indicate that L1-80 is important for the migration of cortical neurons and that the aberrant migration of *reeler* neurons can be partially rescued by the introduction of L1-80.

### Displaced cortical neurons in the *L1*-deficient cerebral cortex

**Figure 6:**
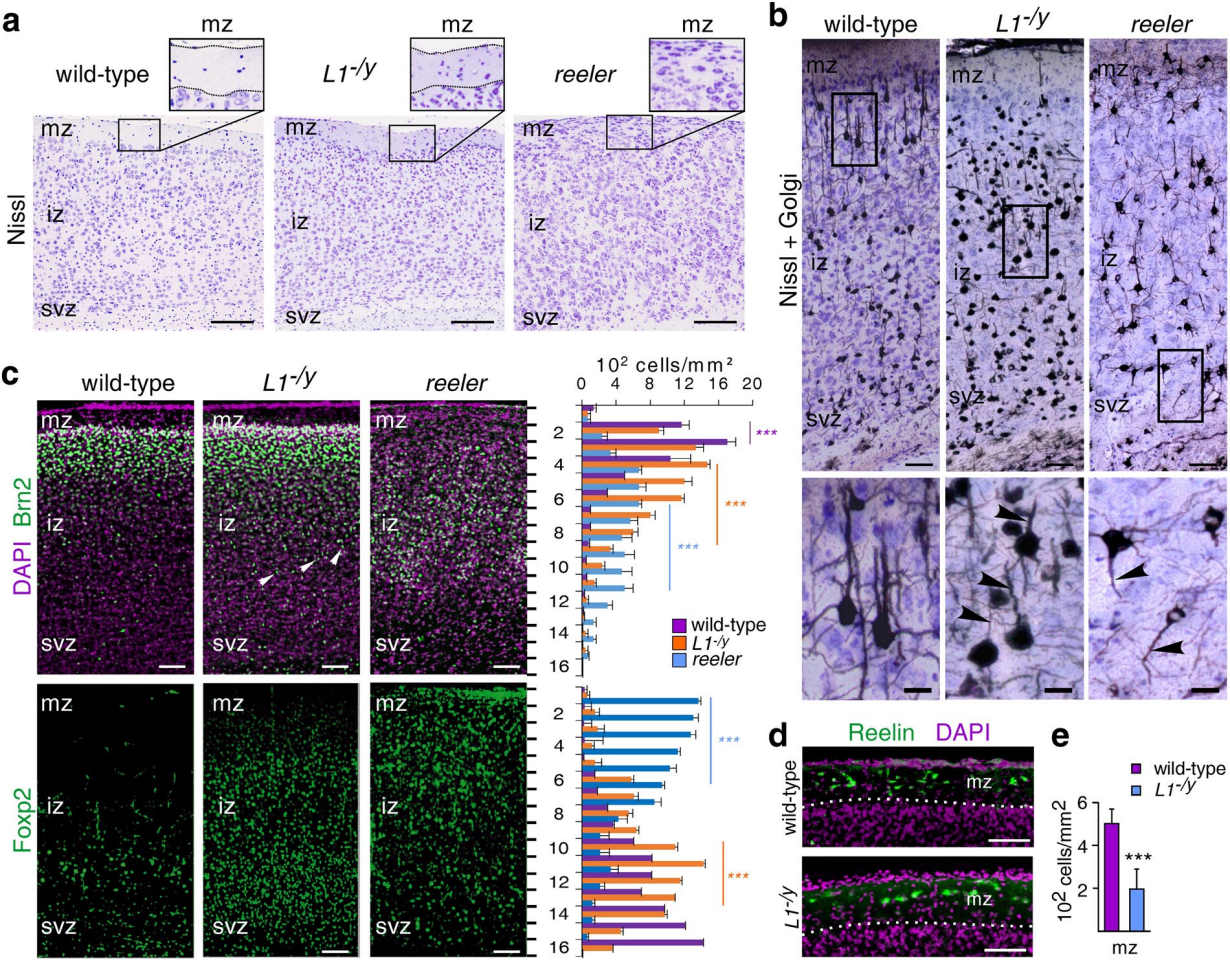
*L1*-deficient mice show deficits in positioning of cortical neurons and reduced number of Cajal-Retzius cells. (**a**) Nissl-staining of cerebral cortices from 6-day-old wild-type and *L1*-deficient (*L1* ^*−/y*^) littermates shows a cell-poor marginal zone (mz) followed by an intermediate zone (iz) and subventricular zone (svz). In contrast, the mz in the cerebral cortex of *reeler* mice contains by more neurons; scale bars: 100 µm. (**b**) Nissl staining combined with Golgi impregnation on cerebral cortical sections from 24-day-old wild-type and *L1* ^*−/y*^ littermates in comparison to *reeler* mice of the same age (low magnification images are shown in the upper panels, scale bars: 50 µm; magnified areas are indicated by rectangles and presented in the lower panels, scale bars: 10 µm). (**c**) The left panels show cerebral cortical sections from wild-type, *L1*-deficient (*L1* ^*−/y*^) and *reeler* mice after immunostaining for Brn2 or Foxp2 (green) in combination with nuclear staining (DAPI, violet). White arrowheads show misplaced neurons; scale bars: 50 µm. The right panels show mean values + SEM for the numbers of Brn2- and Foxp2-positive cells in 16 cortical bins along the mz-svz axis analysing 12 sections of the same area from 4 animals per condition (***p < 0.0001; One-way ANOVA with Tukey’s Multiple Comparison Test; violet asterisks: differences between wild-type and *reeler* neurons; orange asterisks; differences between *L1*-deficient and wild-type neurons; blue asterisks: differences between *reeler* and *L1*-deficient neurons). (**d**) Immunostaining for Reelin (green) using antibody G10 revealed a reduced number of Cajal-Retzius cells in the mz of *L1*-deficient cortex when compared to wild-type littermates. Nuclear staining (DAPI, violet); scale bars: 100 µm. Mean values + SEM are shown for the numbers of Cajal-Retzius cells in the mz analysing 12 sections of the same area from 4 animals per condition (***p < 0.001; Student’s *t-*test).

**Figure 7:**
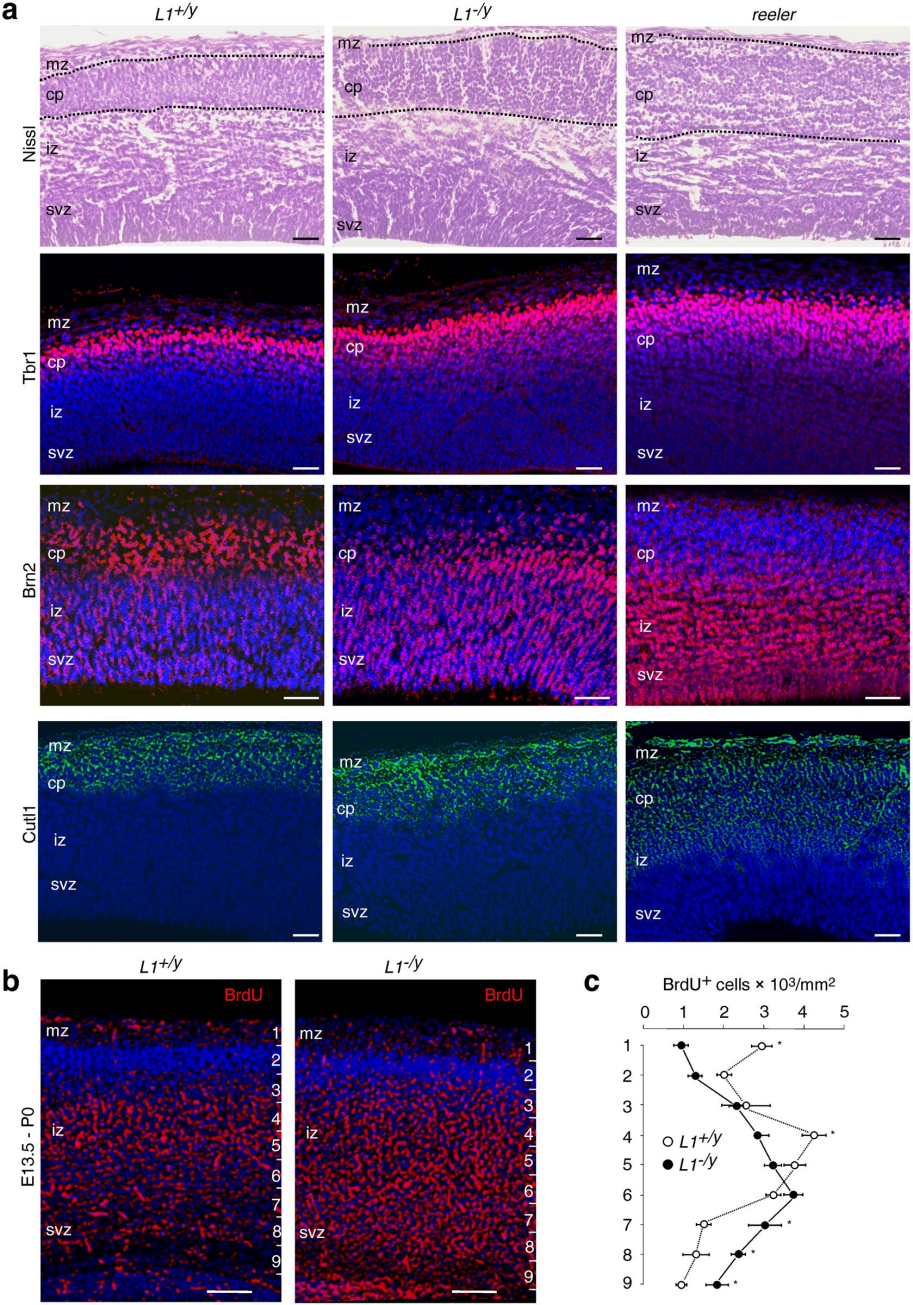
Histological analysis of embryonic wild-type, *L1*-deficient and *reeler* brain cortex sections and BrdU pulse chase labelling. (**a**) Nissl staining and layer-specific immunostaining of cortical sections of *reeler*, *L1*-deficient (*L1* ^*−/y*^) and wild-type (*L1* ^+/*y*^) mice at embryonic day 15; dashed lines indicate the area of the cortical plate (cp). Note that the *L1* ^*−/y*^ cp appeared less compactly developed in comparison to the *L1* ^+/*y*^ cp. The distribution of Tbr1-positive (red), Brn2-positive (red) and Cutl1-positive (green) cells in the *L1* ^*−/y*^ cortex was slightly altered in comparison to the *L1* ^+/*y*^ cerebral cortex, but these abnormalities were not as extensive as the misplacement of cells seen in the *reeler* cortex. Nuclear staining with DAPI (blue); mz, iz, svz: marginal, intermediate and subventricular zones; scale bars: 50 µm. (**b**,**c**) Altered distribution of BrdU-positive cells in the cerebral cortex of *L1* ^*−/y*^ mice. A single BrdU dose (150 mg/kg) was injected intraperitoneally into timed-pregnant females carrying 13.5-day-old L1^+/y^ and L1^−/y^ embryos. Immunohistology for BrdU (red) was performed at birth (P0). The cerebral cortex was divided in 9 equidistant bins and the number of BrdU^+^ cells per mm^2^ was estimated for each bin (right); scale bars: 50 µm. Mean values ± SEM are shown for the numbers of BrdU-positive cells per mm^2^ analysing five sections from three animals per condition (*p < 0.05; Mann-Whitney U-Test).

We next investigated whether *L1*-deficient cerebral cortices show deficits in positioning of cortical neurons and whether these deficits correlate at least partially with those in *reeler* mice. To this aim, sections of wild-type, *L1*-deficient and *reeler* cerebral cortices from 6-day-old mice were Nissl-stained and analysed by light microscopy. In contrast to *reeler* cerebral cortices, the marginal zones in wild-type and *L1*-deficient cerebral cortices appeared to harbour fewer neurons (Fig. 6a). Since Nissl staining does not reveal information on neuronal processes (Fig. 6a), we combined Nissl staining with Golgi impregnation. We found Golgi-impregnated pyramidal neurons uniformly oriented towards the marginal zone in the wild-type cerebral cortex, whereas pyramidal neurons of the *L1*-deficient cerebral cortex occasionally displayed aberrant apical dendrites (Fig. 6b). In the *reeler* cerebral cortex, we noticed many Golgi-positive neurons with misoriented apical dendrites, often pointing towards the subventricular zone (Fig. 6b). Furthermore, we immunostained sections of wild-type, *L1*-deficient and *reeler* cerebral cortices for the layer-specific markers Brn2 (upper layers) and Foxp2 (deep layers). In contrast to wild-type cerebral cortices, many Brn2-positive cells were abnormally found in deep layers, and many Foxp2-positive cells were found in upper layers of *L1*-deficient cortices (Fig. 6c). By contrast, in *reeler* mice Brn2-positive and Foxp2-positive cells were far more dislocated (Fig. 6c). Of note, immunostaining for Reelin revealed a decreased number of Reelin-expressing Cajal-Retzius cells in the marginal zone of cortices from *L1*-deficient mice in comparison to the marginal zone of cortices from wild-type mice (Fig. 6d,e). Additional Nissl staining of embryonic brain sections at an early developmental stage pointed to a less compact cortical plate in *L1*-deficient mice when compared to the dense cortical plate of wild-type littermates (Fig. 7a). Layer-specific immunostainings with Brn2, Cutl1 and Trb1 (superficial/upper layers) as well as a BrdU pulse chase labelling revealed misplaced neurons in the cerebral cortex of *L1*-deficient mice (Fig. 7a–c). Our combined analysis of pre- and postnatal brain development demonstrates that *L1*-deficient cortical neurons are delayed in their migration and have less uniformly orientated apical dendrites. However, these abnormalities were not as pronounced as those in *reeler* mice.

## Discussion

In this study, we show that Reelin and its here newly discovered binding partner and substrate L1 are functionally linked to each other. Both L1 and Reelin are required for neuronal migration and neurite outgrowth. These functions require Reelin’s proteolytic activity, which generates the C-terminal L1-80 fragment by a process that is independent of Reelin’s signalling via ApoER2, VLDLR and Dab1. Concomitantly generated N-terminal extracellular L1 fragment(s) could not be detected in these assays, implying a rapid degradation after generation of such fragment(s). Although the *in vitro* experiments indicate that proteolysis of L1 by Reelin takes place extracellularly, it is conceivable that Reelin cleaves L1 in the intracellular biosynthetic pathway. L1, but not its close homolog CHL1 or the neuronal cell adhesion molecule NCAM, is a substrate of Reelin. Reelin binds to L1 via N-terminal sequences present in full-length Reelin and the Reelin fragments N-R2 and N-R6. It remains to be shown whether other members of the L1 family or, more generally, the immunoglobulin superfamily, are also proteolytic substrate candidates for Reelin.

We have previously described a normal morphology of the cerebral cortex of *L1*-deficient mice based on our analysis of Nissl-stained sections^42–44^. In comparison to 6-day-old wild-type littermates, *L1*-deficient mice did not show obvious changes in cortical lamination following Nissl staining. However, Golgi impregnation and investigation of layer-specific markers in addition to BrdU pulse-chase labelling revealed abnormalities in the positioning and morphology of cortical neurons in *L1*-deficient brains. These novel findings call for a re-interpretation of previous results on neuronal lamination in the *L1*-deficient brain^42–44^ and underscore the importance of L1 for correct brain development. The morphological abnormalities observed in cortices in *L1*-deficient mice partially overlap with those seen in cortices of *reeler* mice, suggesting that the functional interplay between L1 and Reelin and cleavage of L1 by Reelin at early developmental stages controls cerebral cortex development. Indeed, we showed that *reeler* neurons in the cerebral cortex normalised their movement after *in utero* electroporation of L1-80. These findings further support the view that Reelin-mediated L1-cleavage contributes to cortical development. Moreover, our results are consistent with L1 being important for the migration of cerebellar granule cells^1^ and are in agreement with observations on the deficits in *L1*-deficient mice, which are characterized by misorientation of neuronal processes, impaired synaptogenesis and corticospinal tract malformation^45,46^. Notably, silencing of L1 *in utero* has been reported to result in delayed neuronal migration^47^. Migration defects of cortical neurons are known to contribute to mental diseases, such as the L1-syndrome^5,48,49^ or lissencephaly^50^. Thus, our results link proteolysis of L1 by Reelin to neurodevelopmental diseases, highlighting early migratory defects in the pathogenesis of these disorders.

Although some pathological features of the *L1*-deficient mice are also displayed by *reeler* mice, not all abnormalities overlap between both mutants. These findings are understandable under the premise that L1 and Reelin interact with multiple interaction partners that are distinct from each other, and thus are involved not only in overlapping functions, but also in varying pathways leading to diverse phenotypes.

It needs to be pointed out that L1 is cleaved by other proteases at amino acids 840–845 in late developing brain structures^10–12,19^, suggesting even more complex roles of L1 in the maintenance of cortical architecture and function. We here showed that full-length Reelin and its N-terminal fragment N-R6, but not other Reelin fragments, cleave full-length L1 and generate L1-80. Ontogenetic expression studies revealed a close correlation between the expression of N-R6 and L1-80 during the peak phase of neuronal migration, suggesting that both fragments contribute to migration.

Our results shed light on the importance of the N-terminal cleavage of Reelin by proteases, such as ADAMTS-4, to regulate Reelin’s functions. The N-terminal cleavage of Reelin abolishes the protease activity of N-R6, and the originating R3-6 and R3-8 fragments are unable to process L1. Interestingly, introduction of L1-80, but not of full-length L1, into the cerebral cortices of *reeler* mice by *in utero* electroporation partially normalises the migration of cortical neurons. Thus, our results indicate that Reelin’s N-terminus and its central part are required for Reelin’s serine protease activity to generate L1-80 and to promote L1-dependent functions via L1-80.

Since cleavage of the cell surface and transmembrane adhesion molecule L1 is crucial for cerebral cortex development and for directed neuronal motility, it will be important to analyse the molecular mechanisms underlying the directed migration of neurons from the subventricular zone to the marginal zone, when migrating neurons approach the upper cortical layers and detach from radial glial fibers^51^ in the terminal phase of the migratory process^52^. It would be also desirable to investigate whether the mechanisms of directed migration of neurons and their detachment from radial glial fibres depend solely on the L1–Reelin interaction, or whether other molecules determine the “go and stop” phenomena characteristic for neuronal migration in the cerebral cortex. It is conceivable that cytoskeletal elements at the leading edge of neurites extending along radial glial fibres are involved in regulation of migration. Moreover, cognate signal transduction mechanisms via the L1 fragment generated by Reelin could play a role in regulation of migration. Whether these downstream events would depend on the protease activity of Reelin or whether Reelin’s binding to its receptors at the cell surface would be sufficient for downstream signalling will be important to study in the future.

## Methods

### Animals

*Reeler* mice^21^, *ApoER2* ^32^ or *VLDLR* ^53^ single mutant mice, *ApoER2* and *VLDLR* double mutant mice^32^, *Dab1*-deficient mice^30^ and *L1*-deficient mice^7^ were housed at 22 °C on a 12 h light/dark cycle with *ad libitum* access to food and water. All animal experiments were approved by the local authorities of the State of Hamburg (animal permit numbers ORG-679, ORG-604, ORG-578 and TVA 6/14) and conform to the guidelines set by the European Union.

### Antibodies and reagents

Mouse antibody G10 against the N-terminal part of Reelin was from Merck Millipore and mouse Reelin antibody CR50 was a kind gift of André Goffinet (Institute of Neuroscience, University of Louvain, Brussels, Belgium) and Tom Curran (Children’s Mercy Hospital, Kansas City, MO, USA). Goat Brn2 antibody (sc-6029) was from Santa Cruz Biotechnologies (Heidelberg, Germany). Mouse antibody 172 recognising the intracellular L1 domain (anti-CD171; clone 74-5H7; Cat #38101) was from Biolegend (San Diego, CA, USA) and the polyclonal rabbit L1 antibody Anti-L1CAM (ab123990) was from Abcam (Cambridge, UK). Mouse GAPDH antibody (ab9482) and rabbit antibodies against Foxp2 (ab16046), Tbr1 (ab31940), and Cutl1 (ab140042) were from Abcam (Cambridge, UK). Goat CHL1 antibody (AF2147) was from R&D Systems. Production of rabbit NCAM antibodies and of L1-Fc comprising the extracellular part of mouse L1 fused to Fc part of human IgG have been described^54^. Aprotinin, phenylmethylsulfonyl fluoride and the Flag M2 mouse antibody (F1804) were from Sigma-Aldrich (Taufkirchen, Germany). Secondary antibodies coupled to horseradish peroxidase, Cy3 or Cy2 and human Fc were from Dianova (Hamburg, Germany).

CAG-IRES-GFP vector was a kind gift from Melanie Richter and Froylan Calderon de Anda (Center for Molecular Neurobiology, Hamburg). Plasmids carrying full-length Reelin and Reelin fragments N-R2, N-R6, R3-6, R3-8 and R7-8^40^ and purified Reelin^55^ used for ELISA were kind gifts from Tom Curran and André Goffinet. HEK293T cells stably transfected with wild-type Reelin and mock-transfected HEK293T cells^37,56^ were kindly provided by Eckart Förster (Ruhr-University Bochum, Department for Neuroanatomy and Molecular Brain Research, Bochum, Germany).

### Preparation of brain homogenates and Western blot analysis

Brain areas were homogenised in RIPA buffer (20 mM Tris-HCl, pH 7.5, 150 mM NaCl, 1 mM Na_2_EDTA, 1 mM EGTA, 1% Nonidet P-40, 1% sodium deoxycholate, 2.5 mM sodium pyrophosphate, 1 mM β-glycerophosphate, 1 mM Na_3_VO_4_, and 1× cocktail of protease inhibitors (Roche Diagnostics, Mannheim, Germany)) at 4 °C. After centrifugation at 20,000 × g and 4 °C for 10 min, equal amounts of total protein from the supernatants were subjected to Western blot analysis^12^ with Reelin antibodies G10 (diluted 1:1,000), L1 antibody 172 (diluted 1:1,000); CHL1, NCAM or GAPDH antibodies (diluted 1:2,000). Secondary antibodies coupled to horseradish peroxidase were used at a dilution of 1:10,000.

### Tissue preparation, immunohistology and imaging

For paraffin sectioning, fixed brains were incubated in 70% ethanol at 4 °C overnight. Brains were dehydrated in ascending grades of ethanol and cleared in pure xylene. Following immersion in liquid paraffin at 60 °C for 24 h, samples were embedded in paraffin at ambient room temperature. Sections of 10 µm were cut on a microtome (Leica, Wetzlar, Germany) and collected on glass slides. Sections were deparaffinised in pure xylene several times, rehydrated in descending grades of ethanol and immunostained with Reelin antibody G10 (diluted 1:500), Brn2, Foxp2, Tbr1, or Cutl1 antibodies (diluted 1:100). Blocking solution (PBS containing 10% horse serum and 0.01% Triton X-100) was used for dilution of antibodies. Cy3- and Cy2-conjugated secondary donkey antibodies were used at a dilution of 1:500 in PBS. Sections were mounted in Fluoromount containing DAPI (Sigma-Aldrich). Images of immunostained brain sections were taken on a confocal fluorescence microscope (F1000, Olympus, Hamburg, Germany) or a Keyence Fluorescent Microscope (BZ-9000, Keyence, Neu-Isenburg, Germany) and were processed by using the ImageJ software.

### Plasmids and site-directed mutagenesis

Wild-type and mutated L1^10^ were cloned into the CAG-IRES-GFP vector using the In Fusion cloning kit (TaKaRa/Clontech, Saint-Germain-en-Laye, France). For the mutation of Reelin, sequences between the SnaBI and AgeI sites were amplified using the HiFi amplification kit (TaKaRa/Clontech), the plasmid coding for full-length Reelin, the forward primer 5′-AAC CCC ACC TAC GTA CCG GGA CAG GAA TAC-3′ and the reverse primer 5′-GT TAC TGC AAA CCG GTC TCC ATC CGC CTT TCC AAA CAC CAT GGC TG-3′ carrying the mutation (underlined triplet). The plasmid coding for full-length Reelin was digested with SnaBI and AgeI and fused with the amplicon with the mutation using the In Fusion cloning kit.

To amplify sequences coding for L1-80 or for the L1 signal sequence, PCR were performed using murine L1-cDNA^10^ as template and the forward primer 5′‐GAATTC CAT ATC CAC AAA AGC CAC ATA‐3′ containing an EcoRI site and the reverse primer 5′-CTCGAG TTC TAG GGC TAC TGC AGG ATT‐3′ containing a XhoI site or the forward primer 5′‐GAAGCTTTAGCCACCATGGTC ATG GTC GTG ATG CTG CGG TAC‐3′ containing a HindIII site and the reverse primer 5′-GAATTC GAG CAG GCA GGG GCT GCA GAG‐3′ containing an EcoRI site. The amplicons were used for T/A-cloning into pGEM^®^-T-Easy vector (Promega, Mannheim, Germany). Using EcoRI and Xhol restriction sites, the L1-80 amplicon was cloned into pcDNA3 Flag-His A plasmid (Promega) in frame with the Flag-His tag. The signal sequence of L1 was cloned in frame with the L1-80 amplicon via HindIII and EcoRI restriction sites.

### HEK cell transfection, scratch assay, assessment of gap closure and pull-down experiments

Culturing and transfection of HEK293T cells, scratch assay and assessment of gap closure have been described^11^. Serum-free supernatants from transfected HEK cells were dialysed and concentrated 5-fold using 100 kDa cut-off centrifugation columns (Merck Millipore, Darmstadt, Germany) at 4 °C. Pull-down with L1-Fc and Fc using Protein A beads was described^57^.

### ELISA

ELISA (see ref.^12^) was performed as follows: 5 µg of L1-Fc and Fc, 5 µg of serum-free Reelin-containing HEK supernatants and Reelin-devoid HEK supernatants (mock) concentrated 5-fold and dialysed through 100 kDa cut-off Amicon columns, were substrate-coated in 384-well microtiter plates with high binding surface (Corning, Tewksbury, MA, USA) at 4 °C overnight. After washing with PBS, blocking with 1% w/v bovine serum albumin (essentially fatty acid free; PAA Laboratories, Cölbe, Germany) in PBS at ambient room temperature for 1 h, and washing with PBST (PBS with 0.05% Tween 20), 5 µg of L1-Fc or Fc were added to the substrate-coated Reelin and HEK cell supernatants, and 5 µg of purified Reelin were added to the substrate-coated L1-Fc or Fc for incubation at room temperature for 1 h. After washing with PBST, primary mouse monoclonal Reelin antibody G10 followed by an anti-mouse HRP-coupled secondary antibody (Dianova) as well as O-phenylenediamine dihydrochloride (ThermoFisher Scientific, Darmstadt, Germany) as horseradish peroxidase (HRP) substrate were used for detection of Reelin. For detection of L1-Fc and Fc, human anti-Fc-HRP (Dianova, diluted 1:2000) antibody was used. The reaction was terminated by addition of 2.5 M sulphuric acid. Absorbance was measured at 490 nm with the ELISA reader (BioTek, Bad Friedrichshall, Germany).

### Primary cell cultures and explants, treatment with additives, and assessment of neurite length and neuronal migration

Isolation and culturing of murine cerebellar and cortical neurons and explants have been described^11,12,58^. Briefly, cortical and cerebellar neurons as well as explants were treated with 10 µl of HEK cell supernatants per well, 1 µM aprotinin or 2 µl of Reelin antibody CR50 (1 mg/ml) per well for 16 h at 37 °C in a humidified atmosphere containing 5% CO_2_. Neurons and explants were then fixed and stained as described^11,12^. Neurite outgrowth and neuronal cell migration were analysed and quantified by measuring the total neurite length per neuron, the number of cells migrating out of the explant cores, and migration area using a Zeiss Kontron microscope with AxioVision 4.7 software (Carl Zeiss, Jena, Germany).

### Purification of wild-type and mutated Reelin, and L1, proteolytic assay and treatment of explants and hippocampal and cerebral cortex homogenates

Wild-type and mutated Reelin were purified using Heparin High-Trap Sepharose columns (GE Healthcare Life Sciences, Solingen, Germany)^35^. Elution was performed in two steps using 140 mM and 400 mM NaCl, and eluates containing Reelin were dialysed using 100 kDa cut-off centrifugation columns (Amicon). L1 was immunopurified from mouse brains as described^2,11^. For the proteolytic assay, 10 ng of wild-type or mutated Reelin were incubated at 37 °C for 16 h in the absence or presence of 1 µM phenylmethylsulfonyl fluoride with 10 ng of L1 immunoaffinity purified from mouse brain. For the treatment of homogenates, neonatal *reeler* hippocampus or embryonic (E17) *reeler* cerebral cortex was freshly homogenized in PBS and incubated with 100 µl of HEK cell supernatants at 37 °C for 16 h.

### In utero electroporation and histological analysis of electroprorated brains

For *in utero* electroporation, timed-pregnant mice at E13.5 were anaesthetised, the abdominal cavity of the pregnant mice was opened and the uterine horns bearing the embryos were exposed. Thereafter, 2 µg of CAG-IRES-GFP vector coding for wild-type L1, vectors coding for L1-80, N-R2, N-R6, R3-6, R3-8 or R7-8 or empty CAG-IRES-GFP vector pre-mixed with 0.01% Fast Green were unilaterally injected into the ventricle of an embryonic brain. Five electrical square unipolar pulses (amplitude of 30 V, duration of 50 ms, intervals of 950 ms) powered by a BTX electroporation apparatus (model BTX ECM 830; Harvard Apparatus, ThermoFisher Scientific) were applied to the embryos. The uterine horns were placed back into the abdominal cavity. After 60 h, pregnant mice carrying electroporated embryos were sacrificed, the embryos were isolated in ice-cold PBS and killed by decapitation. GFP-positive electroporated brains were collected under a fluorescence microscope and fixed in 4% formaldehyde in 0.1 M sodium cacodylate buffer, pH 7.4, at 4 °C overnight, followed by an overnight immersion in a solution of 15% sucrose in 0.1 M sodium cacodylate buffer, pH 7.4, at 4 °C.

Electroporated brains were embedded in 5% agarose and sectioned on a vibratome (Leica VT1000 S) at 80 µm and immersed in blocking solution (5% normal goat serum and 0.2% Triton-X 100 in 0.1 M PBS), washed, and incubated with an antibody recognising flag-tag (dilution 1:500) at 4 °C overnight. Sections were washed in PBS, incubated with Cy3-conjugated secondary antibody (dilution 1:250) at 4 °C overnight, counterstained with DAPI to label nuclei, mounted in Fluorescence Mounting Medium (Dako, Hamburg, Germany) and subjected to fluorescence microscopy.

### Data Availability

All data generated or analysed during this study are included in this published article (and its Supplementary Information files).

## Electronic supplementary material

Supplementary Figures
