# Supplementary material for: Proteolytic cleavage of transmembrane cell adhesion molecule L1 by extracellular matrix molecule Reelin is important for mouse brain development
Source: Sci Rep. 2017 Nov 10;7:15268. doi: 10.1038/s41598-017-15311-x (PMC5681625; doi:10.1038/s41598-017-15311-x)
Supplement: Supplementary file 1 — Supplementary Figures [file 41598_2017_15311_MOESM1_ESM.pdf]

Supplementary Information

Proteolytic cleavage of transmembrane cell adhesion molecule L1 by extracellular matrix molecule Reelin is important for mouse brain development

David Lutz, Ahmed Sharaf, Dagmar Drexler, Hardeep Kataria, Gerrit Wolters-Eisfeld, Bianka Brunne, Ralf Kleene, Gabriele Loers, Michael Frotscher and Melitta Schachner

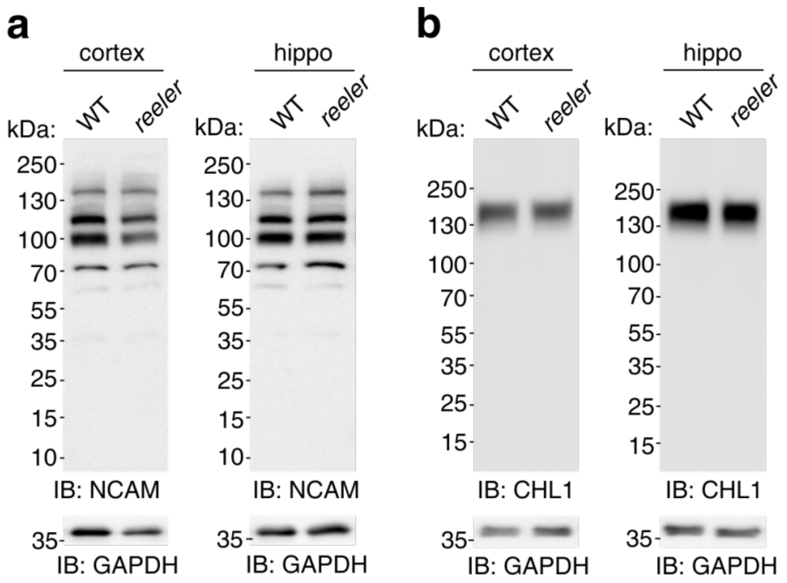

Figure S1. Protein patterns of NCAM and CHL1 are similar in wild-type and *reeler* mice. Immunoblot analysis of homogenates from cortex and hippocampus (hippo) from neonatal wild-type (WT) and *reeler* mice with antibodies against the extracellular domain of NCAM (**a**) and CHL1 (**b**) is shown.

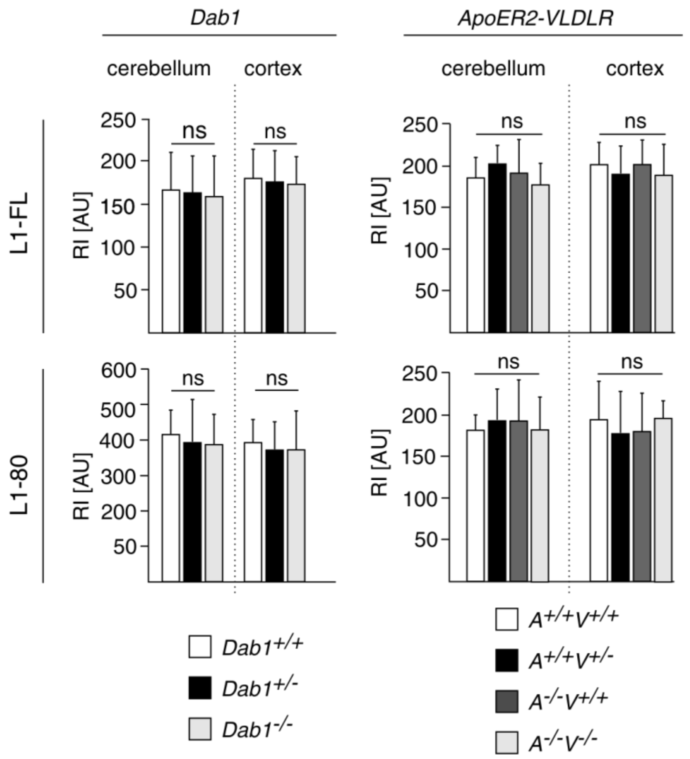

Figure S2. L1 protein patterns are similar in wild-type, *Dab1*-deficient and *ApoER2-VLDLR* double knock-out mice. Quantification of L1-FL and L1-80 levels in cerebellar and cortex homogenates from wild-type, *Dab1*-deficient and *ApoER2-VLDLR* double knock-out mice in comparison to their wild-type and heterozygous littermates is shown.

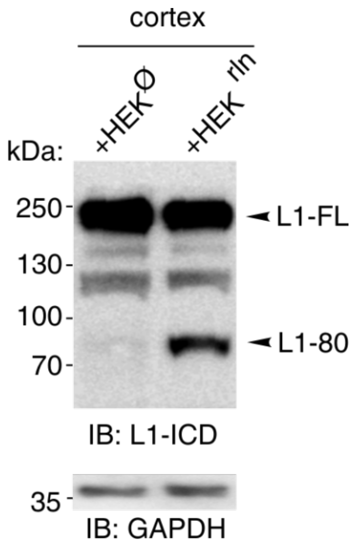

Figure S3. Reelin cleaves L1. Freshly homogenized *reeler* cortex was treated with supernatants from HEK cells expressing (HEK^{rln}) or lacking (HEK^{Ø}) Reelin. Representative immunoblots out of three independent experiments using a polyclonal rabbit antibody against the intracellular L1 domain (L1-ICD) and the GAPDH antibody are shown.

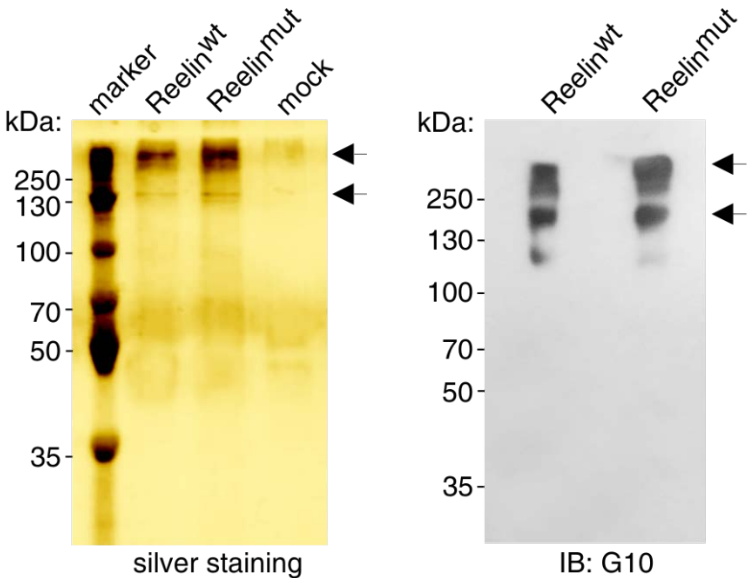

Figure S4. Purification of wild-type and mutated Reelin. Left panel: silver staining (high exposure) showing heparin-purified wild-type (WT) and mutated (S/A-exchange) Reelin fractions (arrows); a mock-purified fraction was used as a negative control. Right panel: immunoblot analysis of heparin-purified wild-type and mutated Reelin fractions using the G10 antibody.
